# Chemo-radiation with or without mandatory split in anal carcinoma: experiences of two institutions and review of the literature

**DOI:** 10.1186/1748-717X-5-36

**Published:** 2010-05-13

**Authors:** Christoph Oehler, Sawyna Provencher, David Donath, Jean-Paul Bahary, Urs M Lütolf, I Frank Ciernik

**Affiliations:** 1 Department of Radiation Oncology, Zurich University Hospital, Zurich, Switzerland; 2 Department of Radiation Oncology, Fleurimont Hospital, Centre Hospitalier Universitaire de Sherbrooke (CHUS), Sherbrooke, Canada; 3 Department of Radiation Oncology, Hôpital de Notre-Dame, Center Hospitalier Universitaire de Montreal (CHUM), Montreal, Canada; 4 Radiation Oncology, Klinikum Dessau, Dessau, Germany; 5 Center for Clinical Research, Zurich University Hospital, Zurich, Switzerland

## Abstract

**Background:**

The split-course schedule of chemo-radiation for anal cancer is controversial.

**Methods:**

Eighty-four patients with invasive anal cancer treated with definitive external beam radiotherapy (RT) with a mandatory split of 12 days (52 patients, Montreal, Canada) or without an intended split (32 patients, Zurich, Switzerland) were reviewed. Total RT doses were 52 Gy (Montreal) or 59.4 Gy (Zurich) given concurrently with 5-FU/MMC.

**Results:**

After a mean follow-up of 40 ± 27 months, overall survival and local tumor control at 5 years were 57% and 78% (Zurich) compared to 67% and 82% (Montreal), respectively. Split duration of patients with or without local relapse was 15 ± 7 d vs. 14 ± 7 d (Montreal, NS) and 11 ± 11 d vs. 5 ± 7 d (Zurich; P < 0.001). Patients from Zurich with prolonged treatment interruption (≥ 7 d) had impaired cancer-specific survival compared with patients with only minor interruption (<7 d) (*P *= 0.06). Bowel toxicity was associated with prolonged RT (*P *= 0.03) duration as well as increased relapse probability (*P *= 0.05). Skin toxicity correlated with institution and was found in 79% (Montreal) and 28% (Zurich) (*P *< 0.0001).

**Conclusions:**

**The study design did not allow demonstrating a clear difference in efficacy between the treatment regimens with or without short mandatory split**. Cause-specific outcome appears to be impaired by unplanned prolonged interruption.

## Introduction

Sphincter-sparing radiotherapy (RT) alone or chemoradiation (CRT) with fluorouracil (5-FU) and mitomycin-C (MMC) is the standard of care for curative treatment of squamous cell carcinoma of the anal canal [1-5]. The Radiation Therapy Oncology Group (RTOG) experience with chemoradiation for advanced stage anal cancer has shown a local failure rate of 20% to 30% with radiotherapy doses of 45 to 50 Gy [2]. Increasing the radiotherapy dose to 59.4 Gy did not appear to increase local control when given in split-course fashion [6].

Concerns about an incorporation of a split in the chemoradiation for squamous cancer have been expressed for years because prolonged RT duration is a known adverse prognostic factor [2,6,7]. In the last few years some institutions have started to omit the mandatory split completely for high-dose RT above 50 Gy in anal cancer [8-11]. Feasibility data have been inconsistent and the recent RTOG 92-08 trial which evaluated 59.4 Gy without mandatory split demonstrated comparable or favourable survival and tumor control compared with split-regimen [8,10,11]. Currently there is no standard in terms of mandatory split and it is unclear whether continuous CRT should be recommended as standard of care for the treatment of anal cancer.

The aim of this analysis was to retrospectively compare the outcome after modern high-dose EBRT with concurrent chemotherapy with or without mandatory split as treated at two independent institutions. We further investigated the feasibility of 3D-CRT (59.4 Gy) without planned split as suggested by the RTOG, reasons for discontinuation and the outcome of the patients with adherence to continuous treatment.

## Patients and Methods

Between 1988 and 2006 84 consecutive HIV-negative patients presenting with histologically proven carcinoma of the anal canal were treated with curative EBRT ± CT at the Zurich University Hospital, Switzerland and the Centre Hospitalier Universitaire de Montreal, Canada. Ninety-nine percent of the patients had squamous cell carcinoma of the anal canal (SCCAC). Clinical characteristics, pattern of care and outcome were analyzed retrospectively by reviewing medical records and interviews of patients after internal board approval.

Pre-treatment staging according to the American Joint Committee on Cancer and the Union International Contre le Cancer (UICC) included digital examination, endoluminal ultrasound or rectoscopy, chest x-rays and either an abdominal ultrasound or CT scanning. Post-treatment evaluation included digital palpation at each visit and regular anal ultrasounds. Anoscopy with post-treatment biopsies and CT or MR scan were performed when a suspicious lesion was identified. The common terminology criteria for adverse events v3.0 was used for scoring acute and late treatment toxicity. Sphincter function was assessed by digital palpation.

3-D conformal RT (6-, 10-, or 18-MV) was applied via a 4-field plan, a dorso-lateral 3-field plan (usually excluding groins) or an AP/PA 2-field plan with electron fields to the groins to the whole pelvis to a dose of 45 Gy/1.8 Gy per fraction (Zurich) or via AP/PA opposed fields to a dose of 24 Gy/2 Gy per fraction (Montreal) using prone or supine position. All patients received an external beam radiotherapy (EBRT) photon boost to the macroscopic tumor region which was delivered via a 2-, 3- or 4-field plan to achieve a total dose of 59.4 Gy (Zurich) or 52 Gy (Montreal). A split of 12 days was intended after whole pelvis irradiation in Montreal whereas no split was intended in Zurich. In Zurich patients developing grade III/IV toxicities (CTC v3.0) treatment was interrupted until side effects resolved. Patients who received a brachytherapy boost in Zurich were not included in the analysis [12]. In Zurich, an EBRT boost was applied to patients who objected an interstitial boost or whose tumor size did not qualify for brachytherapy after 45 Gy EBRT. In Zurich, patients received groin irradiation only if clinically positive (63%) whereas in Montreal, all but one patient (98%) with negative inguinal lymph nodes received prophylactic EBRT to the bilateral groins at a median dose of 24 Gy (range 20-30 Gy). No bolus was used in either institution. All patients, except 1 patient who died during treatment (Zurich), completed curative RT.

Chemotherapy was applied to patients with more advanced stage disease (larger T2, T3/4, N+) (Zurich) or all patients (Montreal). Chemotherapy consisted of fluorouracil (5-FU) and mitomycin-C (MMC) or occasionally cisplatin. 5-FU was applied continuously during 5 days at 750 mg/m^2 ^or 4 days at 1000 mg/m^2 ^in week 1 and 4 or 5 (Zurich) or over 5 days at 1000 mg/m^2 ^in the first week of each RT series (Montreal). MMC was given as a bolus twice (10 mg/m^2^) during week 1 and 4 or 5 or once (15 mg/m^2^) during week 1 (Zurich) or twice (10 mg/m^2^) in the first week of each RT series (Montreal). Cisplatin was given IV, during 1 hour infusion, in week 1 and 4 or 5 at a dose of 40 mg/m^2^/1x (Zurich).

### Statistics

Mean values are indicated with standard deviation. Differences between groups on continuous and categorical variables were tested using the Mann-Whitney test and Fisher's exact test, respectively. Survival was calculated from the beginning of RT to the day of death or the date of last follow-up and time-to-recurrence was calculated from the beginning of RT to the day of recurrence or the date of last follow-up. Survival curves for the two groups were plotted according to the Kaplan-Meier method. Differences in survival across the groups were tested using the Log rank (Mantel-Cox) test. Confidence intervals (CI) were calculated using the formula "95% CI = M ± (SE*1.96)". Log rank test was used to analyze the effect of categorical data on risk of recurrence. Linear regression was used to describe the relationship between local control and RT dose of data from the literature.

## Results

### Patients and treatment characteristics

Thirty-two patients with carcinoma of the anal canal were treated in Zurich and 52 patients in Montreal. The 2 cohorts from Zurich and Montreal had similar patient characteristics (Table 1). Patients treated in Zurich were marginally older than patients from Montreal (61 ± 13 y vs. 56 ± 12 y) (*P *= 0.07) and had more nodal positive disease (*P *= 0.01) (Table 1). RT dose was significantly higher (*P *< 0.001) and mean split duration significantly shorter (*P *< 0.001) in patients from Zurich, though mean overall RT duration time was similar (RT duration includes split). In Zurich, 14 patients (44%) had no treatment interruption whereas the other 18 patients (56%) required a split of any duration. MMC-based chemotherapy was applied more frequently in Montreal (98% vs. 78%) (*P *< 0.01).

**Table 1:** Patient characteristics.

patient characteristics	Zurich	Montreal	*P*
	(n = 32)	(n = 52)	
*Host factors*			
Age (years)	61 ± 13	56 ± 12	0.07
gender (% female)	69	62	
			
Anatomical extent tumor size (%)			
T1	13	15	
T2	28	38	
T3	38	21	
T4	22	23	
LN involvement (%)			0.08
N0	43	71	0.01
N1	17	8	
N2	23	10	
N3	17	12	
			
*Treatment-related factors*			
RT (Gy)	59.4 ± 1	52.1 ± 2	< 0.001
RT duration (days)	52 ± 8	50 ± 8	
Split duration (days)	6 ± 8	14 ± 7	< 0.001
Inguinal RT (%)	56	98	<0.001
Chemotherapy (%)	81	100	<0.01
MMC (%)	78	98	<0.01

Patient characteristics of patients treated with external beam radiotherapy at Zurich (n = 32) and Montreal (n = 52). MMC = Mitomycin-C, LN = lymph node, RT = radiotherapy.

### Treatment response and survival

**Figure 1:**
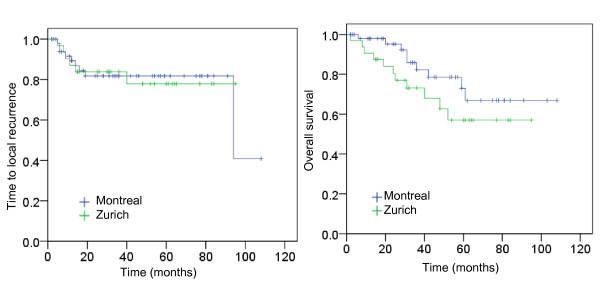
**Cumulative survival of the whole cohort**. Cumulative survival of patients treated at Zurich (n = 32, green line) or Montreal (n = 52, blue line). **1a**: Time-to-local recurrence. Log rank *P *= 0.99 **1b**: Overall survival. Log rank *P *= 0.2.

**Figure 2:**
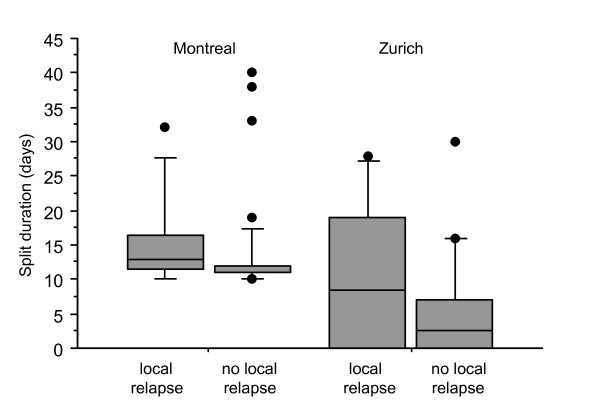
**Box plot analysis of split duration**. Box plot for split duration for Canadian patients with local recurrence (n = 9) or no local recurrence (n = 43) (*P *= NS), and Swiss patients with local recurrence (N = 6) or no local recurrence (n = 26) (*P *< 0.001). The thick line is the median value, the solid box is the interquartile range and the whiskers are the 10^th ^and 90^th ^percentiles, individual cases outside these ranges are plotted.

Curative (chemo-) RT resulted in complete response in 94% of patients at Zurich and Montreal. After a mean follow-up of 40 ± 27 months, there was no difference in overall survival (OS; *P *= 0.2) (Figure 1a) or cancer-specific survival (CSS; *P *= 0.2). The 5-year OS and CSS in patients from Zurich versus Montreal were 57% (95% CI = 37-77%) versus 67% (95% CI = 48-86%) and 74% (95% CI = 57-91%) versus 80% (95% CI = 62-98%), respectively. At 5 years, there was also no difference in local control (78% vs. 82% at 5 y) (Figure 1b) or regional relapse (3% vs. 11%) or distant relapse (17% vs. 8%) between patients treated in Zurich or Montreal. Sphincter-preservation at 5 years was achieved in 74% of patients at Zurich and 79% of patients at Montreal. Split duration of patients with or without local relapse was 15 ± 7 d vs. 14 ± 7 d (Montreal, NS) and 11 ± 11 d vs. 5 ± 7 d (Zurich; P < 0.001) (Figure 2). Overall recurrence probability was associated with advanced T-stage (*P *= 0.06) and N-stage (*P *= 0.09) and increased bowel toxicity (*P *= 0.05) in both cohorts.

In patients from Zurich high-dose (chemo-) radiation of 59.4 Gy was feasible in 14 patients without interruption (44%) and in 4 patients with a split of less than 7 calendar days resulting in 63% with a split of less than 7 calendar days. Reasons for treatment interruption were bowel toxicity (n = 4) (*P *= 0.1), dermatitis (n = 4) (*P *= 0.7), hematological toxicity (n = 2), fistula (n = 2), heart failure (n = 1) or vaginal herpes (n = 1). Univariate analysis of patient characteristics (BMI, nicotine or ethanol) revealed low body mass index (BMI) being predictive for bowel toxicity (P = 0.004) and radiation treatment interruption of any duration (*P *= 0.002). Similar results have been suggested by a previous report [13].

**Figure 3:**
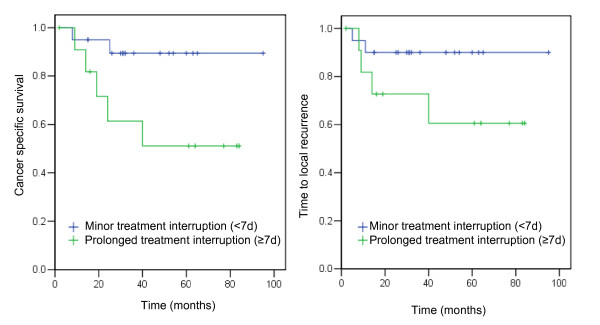
**Cumulative survival of patients from Zurich**. Cumulative survival of patients treated at Zurich with minor treatment interruption (<7 days) (n = 14, blue line) or with prolonged treatment interruption (≥ 7 days) (n = 18, green line). **3a**: Cancer-specific survival. Log rank *P *= 0.06 **3b**: Time-to-local recurrence. Log rank *P *= 0.16.

Patients with prolonged treatment interruption (≥ 7 calendar days) showed impaired CSS (51% vs. 89%; P = 0.03) compared with patients with minor interruption (< 7 d) (Figure 3a). Overall survival (47% vs. 61%; *P *= 0.18), LC (61% vs. 90%; *P *= 0.11) (Figure 3b) and sphincter preservation (61 vs. 83%; *P *= 0.5) did not differ significantly between patients with prolonged (≥ 7 d) and minor (< 7 d) treatment interruptions.

### Treatment toxicity

Acute grade 3/4 toxicity was significantly lower in patients from Zurich (44% vs. 81%; *P *= 0.0002). Seventy-nine percent of patients treated in Montreal experienced dermatitis grade 3/4 compared with 28% of patients in Zurich (*P *< 0.0001). The rate of diarrhea grade 3/4 was similar in the Canadian and Swiss cohorts (4% vs. 13%) as well as chemotherapy-induced hematological toxicity grade 3/4 (15% vs. 4%). One patient from Zurich died due to hematological toxicity. Bowel toxicity correlated with prolonged RT (*P *= 0.03) in univariate analyses.

Chronic toxicity data were available for 66% of patients from Zurich. Thirty-three percent of patients experienced chronic side effects equal to or greater than grade 2: proctitis (40%), incontinence (29%), impaired sphincter tonus (32%) or skin ulceration (5%).

### Review of the literature

**Figure 4:**
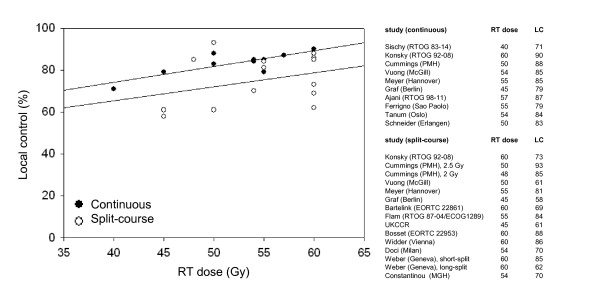
**Review of studies of local control**. Local control rates of studies with or without mandatory split. Linear regression curves (black dots = continuous RT, white dots = split-course RT). RT = radiotherapy, LC = local control.

Of 22 studies identified with primary 3D-CRT and concurrent MMC for treatment of anal cancer (4 prospective randomized, 6 prospective non-randomized, 12 retrospective), data on local control were extracted from 18 studies and were used for regression analysis (Table 2, 3). One study was lacking local control data, 2 studies included split and non-split regimens and for 1 study the updated data were used. Linear regression curves of studies with or without mandatory split demonstrated an increase of local control with higher RT doses (Figure 4). The linear regression curve for local control of studies without mandatory split showed a 10% improved local control through all RT doses compared with studies with mandatory split.

**Table 2:** Review of the literature: prospective trials.

study	n	Stage	total	RT dose (Gy) pelvic	inguinal	Split	CT	OS (%)	LC (%)	CFS (%)	toxicity overall	skin	diarrhea	BM	adherence (%)
**prospective, randomized**															
															
**Ajani (2008)**	324	T2-4	55-59	45	45	cont	MMC, 5-FU	75 (5 y)	87 (5 y)^1^	90 (5 y)	87	48	23	61	
**RTOG 98-11**	320	T2-4	55-59	45	45	cont	Cispl., 5-FU	70 (5 y)	81 (5 y)^1^	81 (5 y)	83	41	24	42	
															
**Flam (1996)**	146	T1-4N0-3	45-50.4	30,6	30.6-45	split (4 w)	MMC, 5-FU	74 (4 y)	84 (4 y)	71 (4 y)	26^2^	7^3^		18	
**RTOG 87-04/ECOG 1289**			(59.4*)		(54*)										
															
**UKCCR (1997)**	283	>T1N0	604	45	e (45)	split (6 w)	MMC, 5-FU	65 (3 y)	61 (3 y)		27	17	5	4	
															
**Bartelink (1997)**	52	T3-4N0-3	60 - 65	45	e (60-65)	split (6 w)	MMC, 5-FU	65 (5 y)	69* (5 y)	71 (5 y)		56	19		
**EORTC**		T1-2N1-3													
															
**prospective, non-randomized**															
															
**John (1996), Konsky (2008)**	20	T1-4N0-3	59,6	30.6 - 45^5^	30.6-45	cont	MMC, 5-FU	85 (5 y)	90 (5 y)	75 (5 y)					80
**RTOG 92-08**	46	T1-4N0-3	59,6	30.6 - 45^5^	30.6-45	split (2 w)	MMC, 5-FU	67 (5 y)	73 (5 y)	58 (5 y)	63	32	9	40*	87
															
**Cummings (1991)**	192	T1-4N0-3	50^6^	50	50	cont	MMC, 5-FU	75	88		75				
**PMH**		T1-4N0-3	50^6^	50	50	split	MMC, 5-FU	65	93		50				
		T1-4N0-3	48	48	48	split	MMC, 5-FU	65	85		36				
															
**Bosset (2003)**	43	T2-4N0-3	59,4	36	e (36)	split (2 w)	MMC, 5-FU	81 (3 y)	88 (3 y)	81 (3 y)		28	12	2	93
**EORTC**		(>4 cm)													
															
**Vuong (2003)**	30	T2-4N0-3	54	27-30	27	cont	MMC, 5-FU	64 (4 y)	91 (4 y)			20	3	13	100
**McGill**															
															
**Schneider (1992)**	46	T0-4N0-3	50 (56-68)^7^	50	50	cont	MMC, 5-FU	84 (5 y)	83 (5 y)	80 (5 y)		35	24	28-35	
**Erlangen**															
															
**Sischy (1989)**	79	T1-4N0-3	40,8	40,8	40,8	cont	MMC, 5-FU	73 (3 y)	71 (3 y)^8^			19	1	3	51
**RTOG**															

EBRT = external beam radiotherapy, RT = radiotherapy, CT = chemotherapy, T = tumor stage, N = nodal stage, w = week, mo = months, y = years, OS = overall survival, LC = local control, CFS = colostomy-free survival, cont = continuous, e = elective, 1 = first event, 2 = grade 4/5, 3 = non-hematological, 4 = alternatively, surgery after 45 Gy, 5 = field reduction after 30.6 Gy from L4/5 to lower sacro-iliac joint, 6 = 2.5 Gy per fraction, 7 = 28% had an EBRT or brachytherapy boost of 6-18 Gy, 8 = locoregional. ***References***: Ajani [1], Flam [2], UKCCR [3], Bartelink [4], John [6], Konsky [8], Cummings [5], Bosset [7], Vuong [9], Schneider [20], Sischy [30]

**Table 3:** Review of the literature: retrospective trials.

study	n	Stage	total	RT dose (Gy) pelvic	inguinal	Split	CT	OS (%)	LC (%)	CFS (%)	toxicity overall	skin	diarrhea	BM	adherence (%)
**Vuong (2007)**	62	T2-4N0-3	54	27-30	27-30	cont	MMC^1^, 5-FU	81	85		37	19	5	13	100
**McGill**	60	T2-4N0-3	45-58.9			split	MMC^1^, 5-FU	54	61		70	43	11	17	
															
**Meyer (2006)**	35	T1-4N0-3	55,8	45	e (45)	cont (≤ 1 w)	MMC, 5-FU	71	85	85		29	3		50
**Hannover**	32	T1-4N0-3	55,8	45	e (45)	split (>1 w)	MMC, 5-FU	63	81	87		27	12		
															
**Graf (2003)**	38	T1-4N0-3	45	30 (45)^2^	30-45	cont	MMC, 5-FU		79						52
**Berlin**	65	T1-4N0-3	45	30 (45)^2^	30-46	split (1 w)	MMC, 5-FU		58						
															
**Tanum (1991,1993)**	117	T1-4N0-3	50 (-54*)	50		cont	MMC, 5-FU	72	75-93			34	9	1	
**Oslo**															
															
**Ferrigno (2005)**	43	T1-4N0-3	55	45	e (55)	cont	MMC, 5-FU	68 (5 y)	79 (5 y)	52 (5 y)		74	44	21	72^3^
**Sao Paolo**															
															
**Widder (2008)**	108	T1-4N0-3	60	30	30^5^	split (2-3 w)	MMC, 5-FU	57	86	51					
**Vienna**	21					or cont^4^									
															
**Doci (1992)**	56	T1-3N0-3	54-60	36	36	split (2 w)	MMC, 5-FU	81 (8 y)	53-74*			5	4	7	
**Milan**															
															
**Ceresoli (1998)**	35	T2-4N0-3	56	45	e (56)	split (2 w)	MMC, 5-FU	71 (5 y)		70 (3 y)		75*		14**	
**Milan**															
															
**Weber (2001)**	45	T1-4N0-3	60^11^	40	40	split (<38 d)	MMC, 5-FU		85^10^						
**Geneva**	45	T1-4N0-3	60^11^	40	40	split (>37 d)	MMC, 5-FU		62^10^						
															
**Constantinou (1997)**	50	T1-4N0-3	54	30-36	30-36 (45)^6^	split	MMC, 5-FU	66 (5 y)	70 (5 y)						
**MGH**															
															
**Mai (2008)**	90	T1-4N0-3	50-54	30.6 (45-50.4)^9^	30-36	cont or split^8^	MMC, 5-FU	86 (5 y)^7^	79 (5 y)			49	1	24	
**Mannheim**															
															
**Grabenbauer (2005)**	87	T1-4N0-3	55.8-66.4	50,4	50,4	cont or split	MMC, 5-FU	75 (5 y)	ca. 90	87 (5 y)		45	34	35	
**Erlangen**															

EBRT = external beam radiotherapy, RT = radiotherapy, CT = chemotherapy, T = tumor stage, N = nodal stage, w = week, mo = months, y = years, OS = overall survival, LC = local control, CFS = colostomy-free survival, cont = continuous, e = elective, 1 = MMC or cisplatin, 2 = T3/4 tumors were treated with 30 Gy to L4/5, T1-4 45 Gy to lower iliosacral joints, 3 = 28% split with 15 d median, 4 = 16% continuous, 5 = 45 Gy if no staging with CT scan, 6 = 45 Gy to medial nodes, 7 = disease-free survival, 8 = 75 pts continuous, 9 pts according to Cummings regimen (48-50 Gy, 4 w split), 6 pts according to RTOG (59.4 Gy, 2 w split), 9 = field reduction from L4/5 to lower iliosacral joint after 30.6 Gy, 10 = locoregional, 11 = brachytherapy boost in some patients. ***References***: Vuong [10], Meyer [11], Graf [16], Tanum [26], Widder [18], Doci [23], Ceresoli [31], Weber [15], Ferrigno [19], Mai [24], Grabenbauer [32], Constantinou [17]

## Discussion

In this retrospective cohort study of 2 institutions comparing modern CRT with or without mandatory split, we found similar overall survival, cancer-specific survival and local control irrespective of split-course regimen. However, different patient characteristics and techniques between the institutions on various levels, and the reluctant use of chemotherapy or prophylactic inguinal RT in many patients in Zurich might have biased treatment outcome. Other limitations of this study are its retrospective character making assessment of toxicity and of cause of death difficult resulting in a relatively low cancer-specific survival. Additionally, patient number was limited and there was a possible treatment bias at Zurich where patients were subjected to either brachytherapy boost or EBRT boost. Adherence to continuous high-dose CRT was feasible in only 44% of patients from Zurich due to severe toxicity such as enteritis, skin or haematological toxicity or fistulae. Bowel toxicity was associated with prolonged RT duration. Skin toxicity was noticed significantly more frequently in patients treated at Montreal. While outcome in terms of tumor control and survival was comparable between patients without or with unplanned interruption, patients with prolonged unplanned treatment interruption (≥ 7 d) in the Zurich group seemed to have worse outcome, particularly cancer-specific survival.

Treatment time and RT-dose, together with chemotherapy, are known prognostic factors in SCCAC [2-5,7,14-18]. Review of the literature revealed 11 studies that evaluated EBRT without prolonged interruption using 3D-CRT and MMC. Two of these studies used RT doses below 50 Gy. Five year local control rates ranged from **79% - 90% **[5,8-11,19,20]. In accordance with the literature, our study demonstrated local control and sphincter preservation rates of 90% and 83%, respectively, at 5 y after continuous (chemo-) radiation with 59.4 Gy (Zurich). Similar results (87%) have also been reported by the most recent RTOG study by Ajani *et al. *using 55-59 Gy/30-32 fractions over intended 5.5 - 6.5 weeks with concurrent MMC [1]. While some studies which compared RT with or without split were unable to find a difference between groups, others showed favorable results or a significant improvement in local control for patients without prolonged unplanned interruption [8,10,11]. In accordance with our observations, Weber *et al. *reported that patients with long unplanned treatment interruption had a significantly worse outcome than patients with short interruption [15]. As shown in Figure 4, cohorts with no major treatment interruption were more likely to have a better local control than cohorts with the same total RT dose but using split-course or interrupted regimens (resulting in a lower biological RT dose). However, some studies with mandatory split regimens also demonstrated excellent local control rates [2,5,7,11,18]. Nevertheless, a majority of trials demonstrated impaired local control for interrupted regimens. Data from one comparative study on dose has been published in abstract form (ACCORD 03). Although no details have been provided on treatment interruptions, doses exceeding 60 Gy do not seem beneficiary [21].

An important feature in this study was the suboptimal adherence to continuous CRT of 44% because of needed treatment interruption due to side effects. Similar results have been reported by Meyer *et al. *(49% > 8 d) [11]. Konski *et al. *reported minor deviation from protocol in 20% of patients [8]. Reasons for treatment interruption in our study were predominantly gastrointestinal toxicity (30%), followed by dermatitis, fistula, heart failure or vaginal herpes. Additionally, a severe dermatitis rate of 80% also hampered adherence to planned short split-course RT in patients treated at Montreal. It is unclear whether this high rate of documented skin toxicity was caused by field size (inclusion of the groins) or due to subject interpretation. On the other hand, another study by Vuong *et al. *demonstrated an adherence rate to continuous high-dose RT of even 100% (Table 4) [10]. In contrast to our study and the one by Meyer *et al*., they applied only 27-30 Gy instead of 45 Gy to the whole pelvis, resulting in lower bowel and hematological toxicity. Interestingly, the same group reported recently that using IMRT instead of conventional 3D-CRT resulted in increased hematological side effects due to bone marrow dose and treatment interruption of 1-3 weeks in 24% of patients [22]. The current RTOG 0529 phase II trial is evaluating adverse events from dose-painted IMRT + 5-FU/MMC compared to the RT+ 5-FU/MMC arm from RTOG 9811.

**Table 4:** Review of the literature: trials using IMRT.

**study**	**n**	**Stage**	**Technique**	**total**	**RT dose (Gy) pelvic**	**inguinal**	**Split**	**CT**	**OS (%)**	**LC (%)**	**CFS (%)**	**toxicity overall**	**skin**	**diarrhea**	**BM**	**adherence (%)**
**Vuong (ASCO 2008)**	26		IMRT	54-59.4	30	30	cont	MMC, 5-FU		71 (1 y)			23	4	42	76
**McGill**	40		3d-CRT	54-59.4	30	30	cont	MMC, 5-FU		85 (1 y)			19	3	18	93
																
**Salama (2007)**	53^2^	T1-4N0-3	IMRT	51,5	45	45	cont	MMC, 5-FU	93 (18 mo)	84 (18 mo)	84 (18 mo)		38	15		58^1^
**Chicago**																
																
**Milano (2005)**	17	T2-4N0-3	IMRT	54-59.4	45	45	cont	MMC, 5-FU	91 (2 y)		82 (2 y)		0	0	53	100
**Chicago**																

IMRT = intensity-modulated radiotherapy, EBRT = external beam radiotherapy, RT = radiotherapy, CT = chemotherapy, T = tumor stage, N = nodal stage, w = week, mo = months, y = years, OS = overall survival, LC = local control, CFS = colostomy-free survival, cont = continuous, e = elective, 1 = median 4 d interruption, 2 = 15% HIV-positive. ***References***: Vuong [22], Salama [33], Milano [34]

Elective groin irradiation is controversial. While in North America, prophylactic inguinal irradiation is a routine practice and the RTOG protocols recommend 30.6 Gy in 17 fractions to this area, in Europe, no elective inguinal irradiation is widely applied [7]. The optimal RT dose for prophylactic iliac lymph node irradiation is also unclear. If RT is given together with CT, particularly MMC, 30 - 36 Gy instead of 45 Gy have been used in many trials [2,8,10,16-18,23,24]. Pelvic relapse has not been consistently reported but seems to be rather low [10,25]. Similarly low inguinal failure rates have been reported after CRT including prophylactic groin irradiation by Das *et al. *(4%) or others [25,26]. However, inguinal failure was also reported to be uncommon (10%) without elective inguinal RT [7,27]. Staging with FDG-PET and sentinel lymph node biopsy (SLNB) are still investigational but might be helpful in the near future [28,29].

## Conclusions

In this retrospective analysis of two cohorts treated to two different institutional guidelines, mainly differing in the standard use of a mandatory split, efficacy of chemoradiation seemed comparable. However, cause-specific outcome may be impaired by unplanned prolonged interruption. Continuous RT may predispose for enhanced gastrointestinal toxicity. Limiting the total dose to organs at risk and field size optimization is likely to improve adherence to treatment and avoid unplanned RT interruptions. Data of the literature point towards improved local control when adherence to continuous or short mandatory split-course CRT with dose escalation is achieved. RT dose escalation to the primary tumor, using IMRT or arc techniques, in combination with IGRT, merit being investigated, in parallel to other treatment modalities such as combination of MMC with cisplatin, novel agents, induction chemotherapy or consolidative chemotherapy.

## Competing interests

The authors declare that they have no competing interests.

## Authors' contributions

CO carried out conception and design, collection and assembly of data, data analysis, manuscript writing, SP carried out collection and assembly of data, manuscript writing, data analysis and interpretation, DD carried out data analysis and interpretation, manuscript writing, JPB manuscript writing, UML carried out data analysis and interpretation, manuscript writing, IFJ carried out conception and design, financial support, data analysis and interpretation, manuscript writing. All authors read and approved the final manuscript.
